# Consideration of the radiation dose delivered away from the treatment field to patients in radiotherapy

**DOI:** 10.4103/0971-6203.79686

**Published:** 2011

**Authors:** Michael L. Taylor, Tomas Kron

**Affiliations:** 1 School of Applied Sciences, RMIT University, Melbourne, Australia; 2 Physical Sciences, Peter MacCallum Cancer Centre, Melbourne, Australia

**Keywords:** Monte Carlo calculations, out-of-field dose, radiation protection, radiotherapy, secondary cancers

## Abstract

Radiation delivery to cancer patients for radiotherapy is invariably accompanied by unwanted radiation to other parts of the patient’s body. Traditionally, considerable effort has been made to calculate and measure the radiation dose to the target as well as to nearby critical structures. Only recently has attention been focused also on the relatively low doses that exist far from the primary radiation beams. In several clinical scenarios, such doses have been associated with cardiac toxicity as well as an increased risk of secondary cancer induction. Out-of-field dose is a result of leakage and scatter and generally difficult to predict accurately. The present review aims to present existing data, from measurements and calculations, and discuss its implications for radiotherapy.

## Introduction

**Figure 1:**
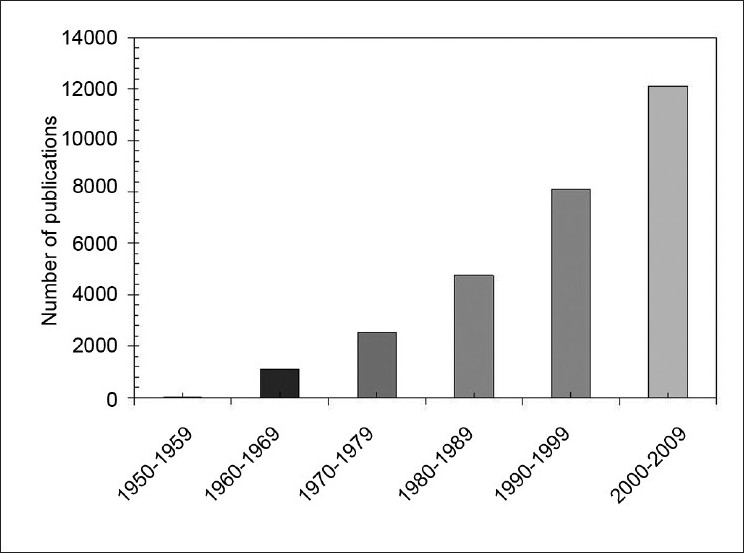
An illustration of the increasing number of publications in radiocarcinogenesis, as reflected by a PubMed search of the terms “radiation-induced cancer,” covering the past six decades

Contemporary external-beam radiotherapy treatment approaches are capable of conforming a focused radiation beam tightly to a defined target volume. Despite the high conformality achievable with modern methods, unwanted doses are nonetheless delivered to untargeted regions of the patient’s body. These doses from outside the primary beam are herein referred to as *out-of-field* doses, which arise from leakage from the medical linear accelerator (linac) treatment head, scatter from collimation devices and scatter from within the patient’s body itself. The increasing efficacy of modern radiotherapy techniques for treatment of cancers is successful in lengthening patients’ lifetimes. The unfortunate corollary of this is that there is therefore longer time in which treatment-induced health complications — such as secondary cancer — may become manifest. Radiation-induced cancer is of increasing clinical interest, as demonstrated by Figure 1.

In this review, an introduction to the mechanisms of, and influences on, radiocarcinogenesis is given, since this is typically considered the gravest potential consequence of out-of-field dose to untargeted organs. A review of studies that have measured or calculated out-of-field doses is also given, followed by a breakdown of individual contributing factors, such as treatment type, linear accelerator type, field size, energy mode and so on. Consideration of these may allow informed selection of equivalently efficacious treatments of the primary tumor that facilitate a reduction in out-of-field dose and associated risks. There have been several interesting reviews published on this topic, including those by Suit, Xu and Tubiana.[1–3]

## Adverse Effects Associated with Low-dose Exposures

While doses delivered outside the field are small relative to the primary-field doses, they are nonetheless of clinical interest because they aregiven to large parts of the body and there is the potential for resultant long-term adverse effects. It is generally accepted that even low doses of ionizing radiation may induce cancer.[4,5] This has been evidenced by documented studies of radiation exposure to populations as a result of war, accidents, occupation or from the diagnosis and treatment of disease. It is not the objective of the present manuscript to review radiocarcinogenesis to any great extent, and the interested reader may find key details of the current scientific understanding of low-dose radiobiological effects in the recent International Commission on Radiological Protection (ICRP) Report 103.[6] There is scientific consensus that the cytotoxic effect of ionizing radiation on cells results from damage to deoxyribonucleic acid (DNA).[7–11] Strands of DNA can be broken directly or indirectly, via interaction with free radicals. The lesions in DNA that result from ionizing radiation include (i) double- or single-strand breaks of the duplex molecule, (ii) chemical alteration of the bases, (iii) chemical alteration of the sugar moieties and (iv) cross-linking to DNA-related matrix proteins or nucleotides in the DNA molecule itself.[12] Single-strand breaks are typically easily and rapidly repaired, whereas double-strand breaks are less readily repaired. The latter can eventuate from the simultaneous scission of both strands close together, or by the interaction of two adjacent single-strand breaks. About 25% of repairs are *misrepairs* in the case of double-strand breaks,[13] depending on the mechanism of repair, and can result in mutations that may ultimately lead to cell death. In the case of damage notresulting in cell death, the daughter cells can carry a radiation-induced mutation. It is generally accepted that unrepaired or misrepaired double-strand breaks are of principal importance in terms of the induction of chromosomal abnormalities and gene mutations.[4]

Such mutation resulting from ionizing radiation is effectively the first stage of the carcinogenic process, known as initiation. The second stage, promotion, involves the acquisition of new properties, such as immortalization, resistance to hypoxia and so on. This comes about by the accumulation of a number of faults in the genome. Subclones can arise from clones of initiated cells in which mutations have occurred. Amongst subclones, there is what Tubiana[14] describes as “Darwinesque” competition, which allows the the subclones that exhibitrapid growth to gain dominance. Ultimately, new subclones emerge with greater autonomy, growing more rapidly, until finally a subclone of cells exists which may proliferate autonomously. Following this stage is progression, in which the cells proliferate frequently despite the absence of stimuli. Cells eventually gain the potential for invasion of peripheral tissues or metastasis.

The low doses to untargeted healthy organs in the human body that occur as a result of scattered and leaked radiation in radiotherapy have the potential to induce cancer (and other health complications) as a result of the treatment. This is a typical stochastic effect whereby the probability of cancer induction is dependent upon the dose whilst the severity is independent. Radiocarcinogenesis is the most serious potential consequence of out-of-field doses. There are several key influences on the risk of radiation-induced carcinogenesis.

**Figure 2:**
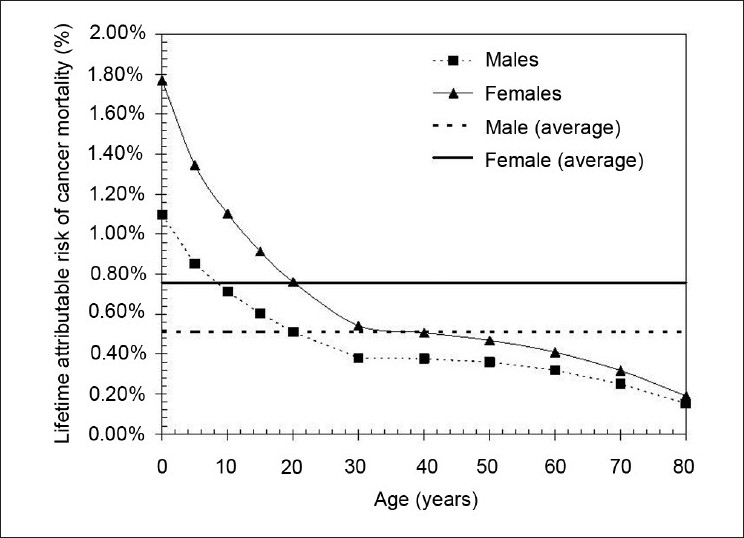
The lifetime attributable risk of cancer mortality (expressed as a percentage of a population exposed to a single 0.1 Gy dose) as a function of age at time of exposure. The data for men (squares) and women (triangles) are shown separately; the average over the 80-year period is 0.76% for women (solid line) and 0.51% for men (dotted line). This figure indicates the age and gender dependency of radiation-induced cancer mortality, constructed using data from the BEIR Report VII (2006)[5]

**Figure 3:**
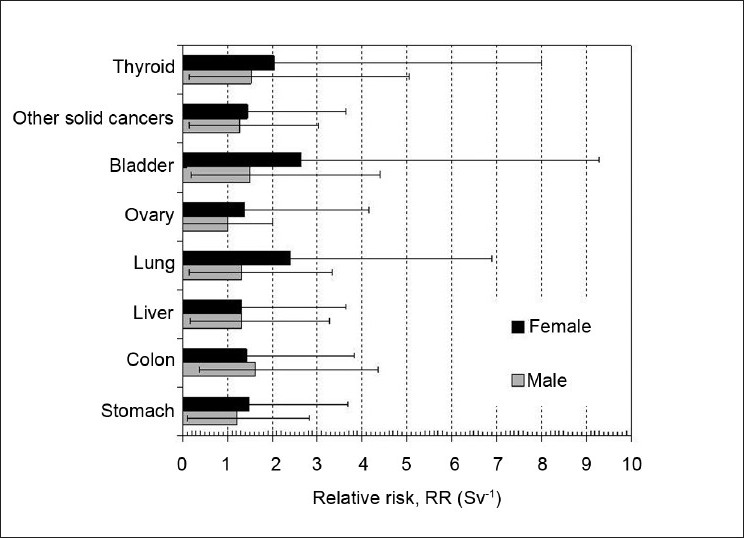
The excess relative risk (ERR) per Sievert for different organ types, corresponding to exposure at age 30 and attained age 60. The ERR is defined as the rate of disease in an exposed population divided by the rate of disease in an unexposed population, minus 1. This figure is based on BEIR[5] data, and indicates the varying sensitivities of different organs to ionizing radiation and the relative sensitivity of females compared to males

One factor is age. There is a higher risk at younger ages of irradiation,[5] as shown in Figure 2. Furthermore, Figure 2 also shows that females are more susceptible to radiation-induced carcinogenesis than males. The average lifetime attributable risk of cancer mortality is 0.76% for women and 0.51% for men (for a 0.1 Gy dose). Time since the inception of irradiation is another key factor. Generally accepted as being of single-cell origin, the development of cancer occurs as a result of successive mutations and extensive proliferation. The development of cancer relies on the unregulated proliferation of mutated cells that are not removed over time via apoptosis or immune system action. A clinically-diagnosed cancer will typically be constituted by several billion cancerous cells. The small probability of particular mutations and the accumulation thereof means process of radiation-induced carcinogenesis is very slow. For solid cancers, the latency is of the order of decades,[15] with the exception of sarcomas (latency may be <10 years) and leukemia (latency may be <5 years).[16] Different tissues exhibit different degrees of susceptibility to cancer induction. Figure 3 highlights the varying sensitivities of different organs; based on Biological Effects of Ionizing Radiation (BEIR)[5] data, the relative risk is given for cancer of the stomach, colon, liver, lung, ovary, bladder and other solid cancers.

**Figure 4:**
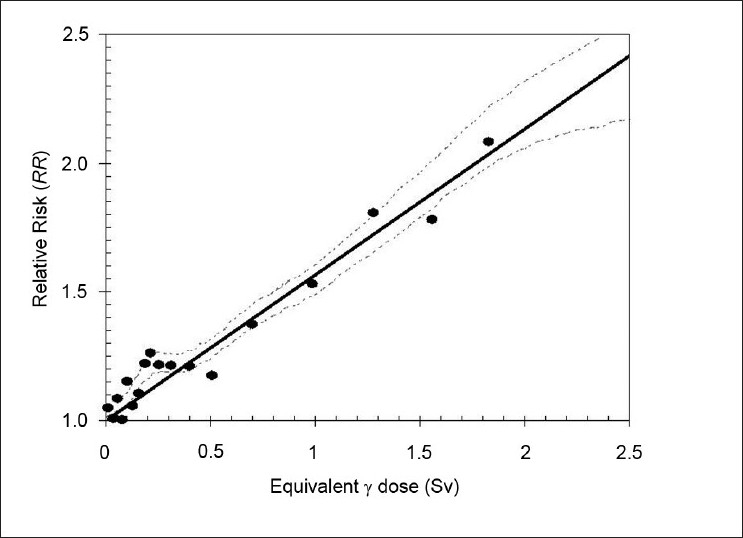
Illustration of the relationship between dose and relative risk, based on data from the atomic bomb survivor cohort; adapted from Pierce and Preston (2000).[21] Note in particular that the extension to low doses (<0.5 Sv), whilst consistent with a linear fit, appears to underestimate the relative risk

Obviously, another key factor affecting the incidence of secondary cancer is dose. The relationship between dose and radiocarcinogenesis is typically considered for dose regimes in two ranges: 0-2 Sv and >2 Sv. The linear no-threshold (LNT) dose-response relationship is generally accepted,[4–6,17] and while there is some argument for nonlinear behavior (see, for instance, Tubiana and Aurengo),[18] ultimately the only generic approach to radiation protection that can be broadly implemented at the present time is the LNT approach.[19,20] Figure 4 illustrates this relationship.

To illustrate the relevant parameters considered in risk calculations, the BEIR VII-preferred risk model for solid cancer induction is shown below. For low doses, a linear fit is suitable, such as

$$I_{D}\;=\;I{\;}_{n}\;\left[1+\left(ERR\right)\left(D\right)\right]$$

where *I_D_* is the rate of incidence, *I_n_* is the background rate (at zero dose), *D* is the dose in Svand *ERR* is the excess relative risk per Sv, given by

$$\mathrm{ERR}\;=\;\alpha_{s}{a_{e}}^{\gamma e^{*}}\;{\left(\frac{t_{a}}{60}\right)}^{\eta }$$

where

α_s_ is a sex-specific risk coefficient, which corresponds to the ERR for exposure at age 30 and 60 years attained age;

a_e_ is the attained age (in years);

γ is the per-decade increase in the age at exposure over the range 0-30 years;

η is the exponent of attained age;

a_e_ is the age at exposure (in years); and

(6.28) $$e^{*}\;=\;\left\{\begin{array}{c} \frac{1}{10}\left(a_{e}\;-30\right),\\ 0 \end{array}\begin{array}{c} a_{e}\;<30\\ ,a_{e}\;\geq 30 \end{array}\right.$$

This is an appropriate risk model for the calculation of site-specific cancer risks using the relative risk coefficients given in the BEIR[5] report. However, it should be noted that there are different preferred models for leukemia and breast and thyroid cancers.

There is also a link between radiation exposure and non-cancer disease mortality. This is discussed in detail elsewhere.[22] Preston *et al*.[21] showed that a linear fit for the dose response is a suitable model, with the linear-quadratic model not fitting significantly better. Table 1 shows the cause-specific excess relative risk per Sv for mortality from non-cancer diseases, based on data from the atomic bomb cohort. All this demonstrates statistically significant risks — thus we cannot neglect out-of-field doses.

## Measurement and Calculation of Out-of-field Dose

### 

#### Overview

**Figure 5:**
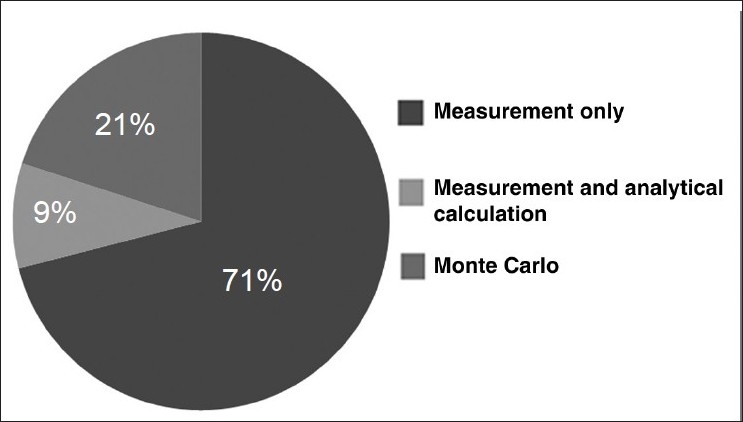
A representation of the percentage of studies (out of 56 selected publications) that have undertaken measurement or calculation (analytical or Monte Carlo) of out-of-field dose

There have been a large number of studies over the past several decades that have investigated out-of-field doses. The majority of these involve measurement of such doses, but many also make calculations of such doses using Monte Carlo radiation transport simulation, analytical approaches, or combinations of these, as indicated in Figure 5. These studies are discussed in two subsections: the first devoted to conventional radiotherapy techniques; the second, to more contemporary methods (such as intensity-modulated radiation therapy) and small-field techniques such as stereotactic radiotherapy.

#### Conventional techniques

There has been increasing strong interest in the out-of-field radiation doses from different treatment units since about the 1970s. The focus of the studies is typically either dose from an occupational radiation safety perspective (particularly in early works; and for ^60^Co sources, where leakage is inevitable) or doses to untargeted critical structures in the patient’s body.

Fraass and Van de Geijn[24] investigated the peripheral dose for a ^60^Co beam, as well as 4-MV, 6-MV and 8-MV photon beams. Doses were reported for water tank measurements for multiple field sizes at a range of distances from the field edge. Transmission and in-patient scatter were separated, which were found to be of similar magnitude. Thermoluminescent dosimetry (TLD) was also performed during treatment of patients. Kase *et al*.[25] similarly studied a ^60^Co beam, as well as 4-MV and 8-MV photon beams. Kase *et al*. also attempted to differentiate head-leakage and scattered radiation, finding that collimator scatter may contribute up to about 40% of the dose outside the treatment field. Francois *et al*.[26] parameterized dose distributions for different beam energies as a function of depth, distance from the edge, field size and shape. An algorithm was thus developed to determine the dose to organs outside the beam at a distance of 10-50 cm from the field edge. The measurements were undertaken with TLD in an anthropomorphic phantom. Measurements were also taken in a large water phantom for the various fields. Limited Monte Carlo calculations were also performed. The American Association of Physicists in Medicine (AAPM) report TG-36[27] reported fetal doses in pregnant women treated with radiotherapy, for a range of delivery conditions. Van der Giessen[28] measured doses in a water phantom for 4 Cobalt machines and 37 linear accelerators to investigate variation in peripheral doses amongst machines from 7 different manufacturers. Variation of leakage radiation dose was found to be small amongst the varying designs; however, collimator dose was found to vary up to 50%, depending on the collimator / flattening filter design. In his PhD thesis, Van der Giessen[29] provides results from studies of various machines (with a focus on ^60^Co), mostly using water phantoms to collect data or by evaluation of published data and leakage / collimator scatter data provided by other clinics / institutions. Dose was also measured on patients’ perinea using TLD. The studies constituting his thesis were published separately as articles, mostly in *Int. J. Radiat. Oncol. Biol. Phys*.[28,30–33]

Broadly, regarding photon doses outside the treatment volume for ‘classical’ methods, one may conclude that the photon dose decreases with decreasing field size and drops approximately exponentially away from the field edge, and neutron doses are more dependent on beam energy than distance from the field edge.

#### Intensity-modulated radiotherapy and small-field delivery

The advent of intensity-modulated radiotherapy (IMRT) has given rise to concerns over the fact that the total number of monitor units used is often greater than that for treatments for equivalent cases using, for instance, three-dimensional (3D) conformal radiotherapy. The additional monitor units may result in additional leakage dose and thus increase the dose to untargeted critical structures. Contemporary IMRT delivery is typically undertaken with multileaf collimators (MLCs) or mini-multileaf collimators (MMLCs) replacing, or attached as, tertiary / quaternary collimators on a linear accelerator. Many of the works discussed here involve measurements to investigate the influence of the MLC on out-of-field doses, also in the specific context of IMRT. Note that the focus of the discussion is on peripheral photon doses.

Followill *et al*.[34] undertook a study of doses outside the treatment fields for IMRT with 6-MV, 18-MV and 25-MV beams, for which the photon whole-body equivalent doses per cGy were 80 µSv, 6.5 µSv and 10 µSv, respectively. The respective neutron doses were 0.0 µSv, 46 µSv and 76 µSv. Using risk values recommended by the National Council on Radiation Protection and Measurements (NCRP), they calculated worst-case scenario risks of cancers to be between a minimum of 0.4% (for a conventional nonwedged 6-MV beam) and 24.4% (for a 25-MV tomotherapy beam). Stern[35] investigated whether the presence of an MLC would influence the peripheral dose when positioned at the field edge defined by the jaws. For 6-MV and 18-MV beams at all depths and distances studied, configuring the MLC leaves at the field edge yielded a reduction in peripheral dose of 6% to 50% compared to the MLC leaves fully retracted. In the latter case, peripheral doses matched those for a linac without an MLC. As mentioned earlier, the AAPM report TG-36 can be used to estimate the peripheral dose distributions.[27] Mutic and Klein[36] undertook a number of measurements with an ionization chamber in a water-equivalent plastic phantom with various MLC leaf settings, including full retraction. Peripheral dose distributions with the MLC fully retracted and collimator rotated to 180° were similar to TG-36 data, but lower with MLC field shaping. They also showed that rotating the collimator to 90° with full MLC retraction may reduce the peripheral dose up to a factor of 3 (compared to TG-36).

Chibani and Ma[37] employed Monte Carlo N-Particle eXtended (MCNPX) to study the dose from photon-induced nuclear particles (neutrons, protons and alpha particles). Varian beams are found to produce more particles than the Siemens beams, due to higher primary electron energies. Neutrons are found to contribute more than 75% of the total dose-equivalent ratio. Chibani and Ma compared the model to measurements. The dose-equivalent from leakage neutrons (at 50 cm off-axis distance) represents 1.1%, 1.1% and 2.0% likelihood of fatal secondary cancer from a 70-Gy treatment delivered by the Siemens 18-MV, Varian 15-MV and Varian 18-MV beams, respectively. Vanhavere *et al*.[38] performed measurements in air, at different depths in a plexi-phantom and using a Rando-Alderson phantom for gammas and neutrons with an 18-MV linac. Organ equivalent doses and effective doses (estimated by different methods) were evaluated for a range of organs. For a prostate cancer IMRT treatment, the effective dose (using Rando-Alderson phantom) was found to be about 30 mSv per 2 Gy target dose, 13% of which is attributed to neutrons.

Sharma *et al*.[39] noted that dynamic fields (consisting of constant-width strips moved from one bank to the other) required between 2 and 14 times as many monitor units as static fields to achieve the same dose at isocenter, for various arrangements. Peripheral doses were between 2 and 15 times higher for the dynamic case, depending on field size, etc. They also compared patient-specific intensity-modulated fields with dynamic MLC fields with similar jaw settings and discovered that the two are sufficiently similar to use the dynamic MLC data to predict out-of-field doses for comparable patient-specific cases.[40] Kry *et al*.[41] highlighted that determination of such out-of-field doses requires tedious measurement or calculations that exhibit high uncertainty. They used the MCNPX Monte Carlo code to model a Varian Clinac 2100 operated at 6 MV, modeling dose distributions away from the central axis and measuring dose distributions with an ionization chamber (in a water phantom) and TLD (in an acrylic phantom). In a different publication, Kry *et al*.[42] describe a similar study for 18-MV photons. In the latter work, discussion of neutron dose was also included.

Wiezorek *et al*.[43] performed point dose measurements at different depths in a solid phantom at 29 cm off-axis distance, for a Siemens Oncor Impression linac with energies of 6 and 15 MV. Peripheral doses associated with artificial fluence distributions were compared with open beam contributions. Measurements were performed with two types of TLDs to quantify photon and neutron doses separately. Neutrons were only detected for 15 MV. The photon contribution to peripheral dose increased (compared to open field) when using segmented multileaf modulation (sMLM) for IMRT, and even further when using compensators.

Also of interest are the out-of-field doses from the small fields used in stereotactic radiotherapy (SRT). The doses involved in SRT are generally much higher than those in IMRT treatments, and are delivered in relatively few fractions. (Note that stereotactic *radiosurgery* involves a single fraction only.) Another point worth noting is that patients often receive stereotactic treatment for *non*-malignant lesions — and thus have relatively long potential lifetimes. The out-of-field doses from these high-dose treatments are thus of significant interest.

Ioffe *et al*.[44] quantified the dose rate as a function of distance from the isocenter in a RANDO phantom for Gamma-Knife treatments. Hasanzadeh *et al*.[45] constructed an anthropomorphic phantom and undertook TLD measurements of dose in untargeted organs for Gamma-Knife radiosurgery. Petti *et al*.[46] developed Cyber-Knife plans for a thorax lesion and brain lesion in an anthropomorphic phantom and measured the dose at various depths and distances outside the treatment field using TLD. Peripheral doses were found to be 2 to 5 times higher than those in a comparable Gamma-Knife treatment, and up to 4 times higher than those in an IMRT treatment. The relatively large peripheral dose is attributed to greater leakage of the Cyber-Knife unit. Chuang *et al*.[47] investigated reduction of out-of-field doses from the Cyber-Knife system resulting from a shielding upgrade, with the observation that doses were generally reduced by 20% to 55%.

Maarouf *et al*.[48] examined the radiation exposure of organs at risk and assessed the risk of late effects (such as secondary tumors or hereditary disorders) following stereotactic linac radiosurgery of intracranial tumors. TLDs were placed superficially on patients’ (*n*= 21) eyelids, thyroid, breast and regions of the ovary / testes. The organ receiving the highest doses was the eye lens (276 ± 200 mGy), followed by the thyroid (155 ± 83 mGy), breast (47 ± 22 mGy), ovary (12 mGy) and lastly the testes (9 ± 3 mGy). The absorbed doses thus ranged between 0.025% and 0.76% of the target dose. They recommended the use of conformal beams employing micro-multileaf collimators and avoiding beams directed toward the trunk. Solberg *et al*.[49] compared conventional noncoplanar arc, static field conformal and dynamic arc field shaping approaches to radiosurgery. In terms of peripheral dose, it was found to decrease as additional beams or arc degrees were added with either of the conformal approaches. Ultimately, dynamic arc shaping was found to be preferred for its efficiency and efficacy in delivery of a homogenous dose whilst minimizing peripheral dose, for radiosurgery applications. More recently, Taylor *et al*.[50] investigated out-of-field doses from mini-multileaf collimator-shaped fields, and described a number of simple techniques for the minimization of out-of-field dose and its associated risks. The authors found doses of the order of cGy in out-of-field regions — a substantial dose in radiation protection terms — and observed that simple treatment techniques such as aligning the craniocaudal direction of the patient with the x-plane of the collimator can reduce dose by up to an order of magnitude. The latter result was confirmed in a later study of doses in small-field radiotherapy of pediatric patients, whereby Taylor *et al*.[51] found that doses are, on average, 40% less along the x-plane (compared to the y-plane). Furthermore, the authors also found that far from the primary field, about half the out-of-field dose is due to leakage; that the use of a linac with a bending magnet resulted in dose about 40% higher than the straight waveguide unit; and that coplanar treatments with beams avoiding the trunk of the body can reduce dose to organs at risk by an order of magnitude.

Tomotherapy is another modality of interest in terms of out-of-field doses. Tomotherapy almost invariably involves a larger number of ‘monitor units’ than an equivalent treatment delivered by conventional radiotherapy, with leakage being a major possible source of out-of-field doses delivered to patients.[52,53] There have also been studies on proton beam therapy in relation to out-of-field doses. These methods are not discussed in any greater detail here because the focus of this work is conventional external-beam radiotherapy, which is far more widely employed than the latter methods.

IMRT treatments often require between 3 and 5 times the number of monitor units to deliver (compared to a conventional treatment). Kry *et al*.[54] measured the photon and neutron out-of-field dose equivalents to various organs using different treatment strategies, energies and accelerators. Photon dose decreased exponentially away from primary field; neutron dose was found to be independent of the distance from treatment field. Neutrons contributed significantly to out-of-field dose for *E* > 15 MeV. Considering out-of-field doses, Kry *et al*.[55] found that the maximum risk of fatal secondary malignancy was 1.7% for conventional radiation, 2.1 % for IMRT with 10-MV x-rays and 5.1% for IMRT with 15-MV x-rays. Kry *et al*.[56] also examined the uncertainty in risk estimates relating to out-of-field doses, with the result that risk estimates for secondary malignancy were subject to very large uncertainties. It was shown, however, that it is possible with relatively good accuracy to identify preferable modalities based on the ratio of risk estimates. In a recent study, Ruben *et al*.[57] compared IMRT with three-dimensional Conformal Radiation Therapy (CRT) in terms of carcinogenic risk. Equivalent plans were constructed for prostate, breast and head-and-neck treatments. The risk of radiation-induced malignancies in organs outside the target volume was calculated using two dose-response models for radiocarcinogenesis. Ultimately, the risks were found to be comparable between the two modalities. Depending on the technique and region of interest, risks ranged between 1% and 2% for one risk model, and between 0.5% and 1% for the other model. There is a significant body of literature covering epidemiological studies of cancer induction in radiotherapy patients, an overview of which is given in the subsequent section. Reft *et al*.[58] performed *in vivo* patient and phantom measurements of the secondary out-of-field photon and neutron dose equivalent for 18-MV IMRT treatments. It was found that the photon dose dropped by a factor of two for distances of 10 and 20 cm from the field edge, while the neutron dose remained the same (within experimental uncertainties). There is an indication that 18-MV IMRT results in higher neutron doses (factor of 2 or 3) compared to three dimensional CRT (3DCRT). Klein *et al*.[59] collected peripheral dose data in a phantom at distances ranging from 5 to 72 cm away from the field edges of small (2 to 10 cm) IMRT fields. Micro-ionization and cylindrical ionization chambers were arranged in a phantom representing a 3-year-old child at locations corresponding to the thyroid, breast, ovaries and testes. Distant peripheral dose (dominated by head scatter) was higher than predicted. For example, doses to the testes were 3 to 5 times higher for IMRT compared to conventional treatment.

There are a range of influences on the out-of-field dose, which are detailed in the following section.

#### Contributions to out-of-field dose

Out-of-field dose is essentially the combination of leakage from the accelerator head, scatter from collimators, from within the patient’s body and from the rest of the treatment room. It is possible to reduce out-of-field doses (and corresponding risks to the patient) by careful choice of treatment arrangement. To help facilitate this, the main influences on out-of-field dose are discussed here.

#### The influence of accelerator type

**Figure 6:**
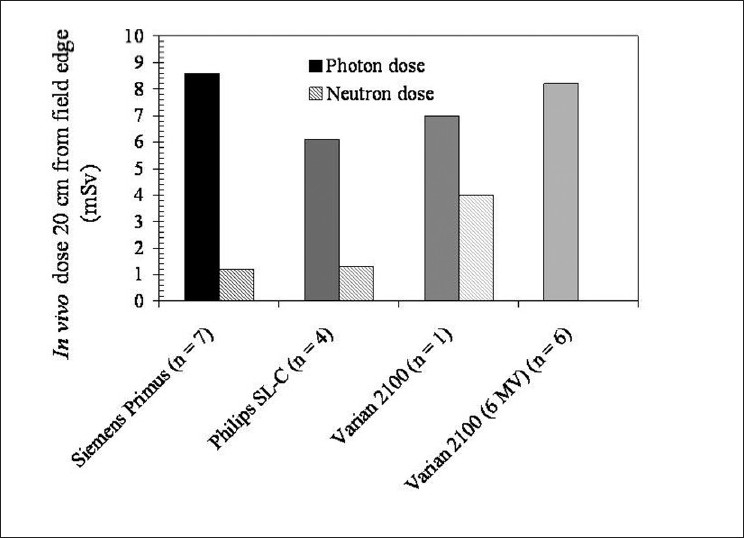
Data from Reft *et al*. (2006)[58] shows the difference between linac models in terms of out-of-field dose for 18-MV IMRT of the prostrate. *In vivo* measurements were undertaken measuring both photon (solid) and neutron (cross-hatched) doses; the data shown here corresponds to doses at a distance of 20 cm from the field edge. Measurements were performed for the same-model accelerator at different centers (reflected by the number n)

Because out-of-field dose to untargeted regions of a patient’s body is a result of a combination of leakage and scatter, it is likely that different linac models (having different shielding designs) will generate different out-of-field doses. Figure 6 illustrates this quite clearly for a Siemens Primus, Varian 2100 and Philips SL-C operated at 18 MV (listed in order of decreasing out-of-field photon dose).

**Figure 7:**
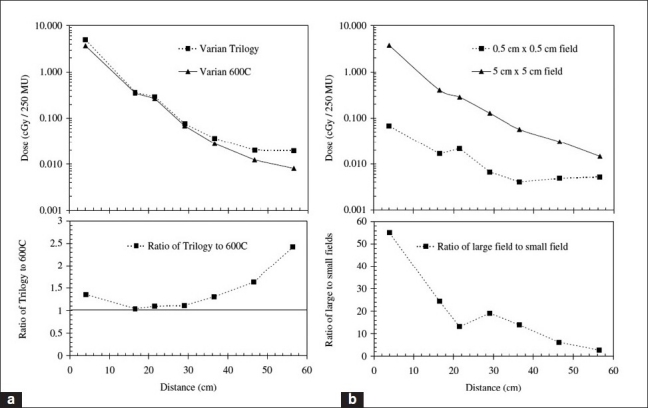
(a) A comparison of out-of-field dose from the (vertical waveguide) Varian 600C with that from the (multi-mode) Varian Trilogy. Note that for the latter linac, the doses are consistently higher than the single-energy mode linac, despite both being operated at the same energy of 6 MV. The differences in out-of-field dose may be attributed to the significant differences in treatment head design; in particular, unlike the 600C, the Trilogy possesses a bending magnet. Data adapted from Taylor *et al*.[51] (b) An indication of the decreasing influence of field size on out-of-field dose (from a Varian Trilogy) with increasing distance from the isocenter. Taking mean organ doses from a study on pediatric radiotherapy out-of-field dose by Taylor *et al*.,[51] it is clear that while differences between large (5 × 5 cm^2^) and small (0.5 × 0.5 cm^2^) fields are significant close to the primary field, further away the field-size–dependent contributions to out-of-field dose (i.e., collimator scatter and patient-scatter) are reduced. In both (a) and (b), the mean values of the published doses are presented, and the horizontal scale is the distance from isocenter

**Figure 8:**
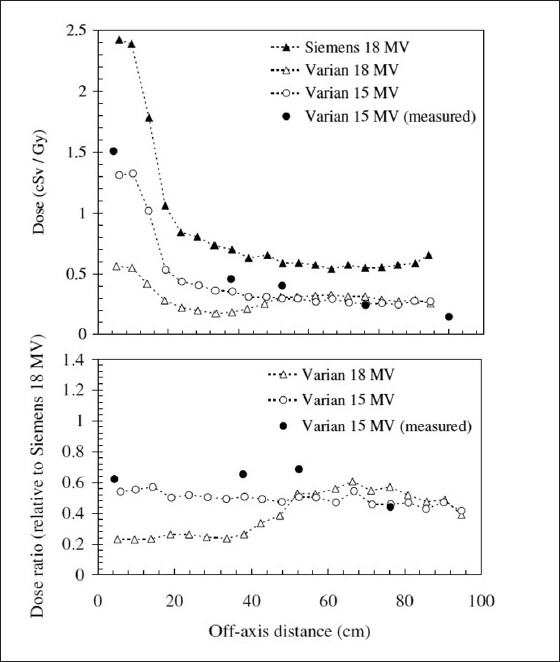
An indication of the variation in neutron dose that exists between operating at 15- and 18-MV modes, and between different linac manufacturers. This data shows MCNPX-calculated neutron doses along the plane of the couch for a Varian 2160C (15 MV and 18 MV) and Siemens Primus (18 MV) (Chibani and Ma 2003). Also shown are several measured data points for the Varian 15 MV. The subplot below the primary figure shows the ratio of these doses to the Siemens 18 MV case

**Figure 9:**
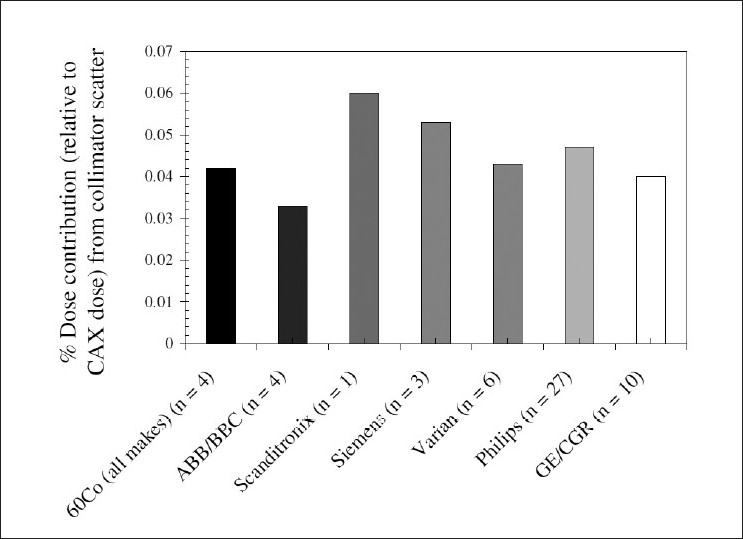
The contribution of out-of-field dose as a result of collimator scatter varies amongst linac designs. This figure shows data adapted from Van der Giessen (1996)[28] indicating this variation. The percentage contribution of collimator-scattered dose (at an off-axis distance of 50 cm) relative to the dose at the central axis is given for seven different linac types. Measurements were taken at different centers with various models; the total number of measurements is given as n in the figure. The doses correspond to a standard field size of 10 × 10 cm^2^

Figure 7(a) also shows very interesting consequences for choice of linac. This data[51] shows that even when operated in the same energy mode (6 MV), there is a significant difference between the out-of-field doses from the (multi-mode) Varian 2100 and the (single-mode) Varian 600C. The dose from the Varian 2100 is up to 250% higher for far out-of-field regions than that from the Varian 600C. This discrepancy is likely to be due to the larger horizontal waveguide of the Varian 2100 and the presence of a bending magnet (the latter acting as a further source of bremsstrahlung). The Varian 600C has a vertical waveguide directed at the isocenter and no bending magnet. Figure 8 illustrates the differences between Siemens and Varian machines, with the latter delivering out-of-field doses only 20% to 50% of those delivered by the Siemens Primus.[37]. Neutron doses are clearly higher with the Varian machine, however [Figure 6]. The different contributions to out-of-field dose from collimator scatter for a range of machines are given in Figure 9. Kry *et al*.[55] found that intensity-modulated radiotherapy (IMRT) in 6-MV mode with Varian and Siemens linacs resulted in risks of fatal secondary cancer of 2.9% and 3.7%, respectively

#### The influence of field size

**Figure 10:**
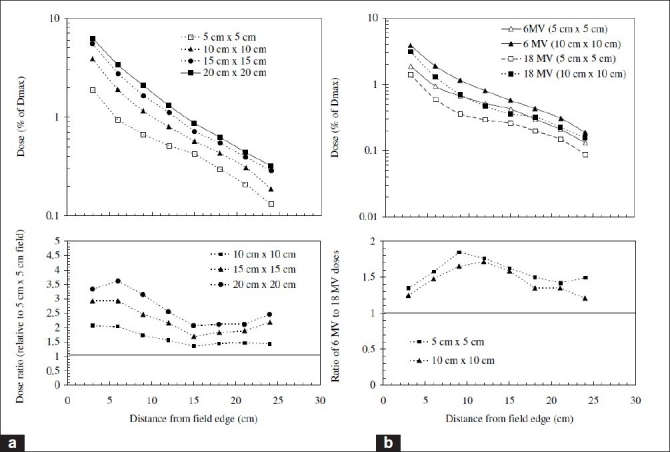
(a) An indication of the variation of out-of-field dose from a Siemens Primus as it varies with field size (shaped with jaws, full MLC retraction). Close to the primary field, the doses from the larger fields are greater, but this difference decreases with increasing distance from the field edge. (b) An illustration of the difference in out-of-field dose depending on energy mode. The out-of-field doses in 6-MV mode are consistently greater than in 18-MV mode. The data shown is adapted from Mazonakis and Zacharopolou[20]

**Figure 11:**
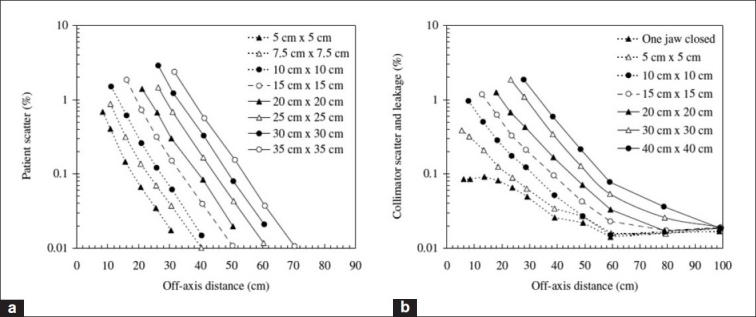
(a) The percentage contribution of patient-scatter to out-of-field dose for a range of field sizes from a 60Co unit (Theratron 780) (Van der Giessen and Hurkmans, 1993).[30] (b) The contribution of collimator scatter and head leakage (as a percentage) to out-of-field dose for a range of field sizes from a 6-MV treatment beam (GE Saturne 41) (Van der Giessen, 1994)[31]

Intuitively, one would expect that for larger field sizes, higherdose would be delivered to out-of-field regions, as a result of increased patient-scatter. Generally this is indeed the case. Taylor *et al*.[51] showed that in the context of linac-based stereotactic radiotherapy, the out-of-field dose tends to (approximately) increase with the increase in side length of the field. For example, a square field of side length 9.8 cm is roughly four times greater than a field of side length 2.4 cm, and the out-of-field dose is about four times greater. However, the difference in out-of-field dose between different field sizes decreases with increasing out-of-field distance. This is made clear from Figure 7(b) — in particular, the subplot showing the ratio of a 5×5-cm^2^ field to a 0.5×0.5-cm^2^ field, which approaches unity with increasing distance from isocenter.[51] A similar result is shown in Figure 8. (Note that Figure 10 shows the distance from field edge, not from isocenter.) This means that at large distances, head leakage is the dominant influence on out-of-field dose. Figure 11 also shows the influence of field size on patient-scatter[30] and collimator scatter and leakage.[31]

#### The influence of beam quality

The energy mode also influences out-of-field dose, as evidenced by Figure 6, Figure 8(b) and Figure 9. Lower-energy beams tend to result in greater out-of-field photon doses than higher-energy modes. This is because lower-energy photons are less forward-scattered than higher-energy photons.[59] As such, one would expect patient-scatter in low-energy modes to result in greater out-of-field dose. One would also expect, however, that this would be pronounced at intermediate distances but less so at far distances, since from the previous section we expect patient-scatter to be less influential far out-of-field. Indeed, from Figure 9, it is clear that the Varian linacs in 15-MV and 18-MV modes generate comparable out-of-field doses at far off-axis distances. The problem with high-energy modes, however, is that the photonuclear effect may generate neutrons that contribute to the out-of-field dose. This is illustrated in Figure 6. It has been shown that neutron doses may not significantly increase the risk of radiocarcinogenesis for IMRT with a linac operated in 18-MV rather than 6-MV mode.[60]

#### The influence of leakage, collimator scatter and patient-scatter

As discussed, the influence of leakage, collimator scatter and patient-scatter may be inferred to some extent by the influence of field size [Figure 7(b)]. A number of authors have made explicit attempts to determine separate influences of these. Figure 10(a) directly indicates that the (percentage of central axis, CAX) dose attributable to patient-scatter decreases with increasing distance.

Van der Giessen[31] treated the collimator scatter and leakage together, and from Figure 10(b) it is clear that the different field sizes converge far from the primary beam, and the contribution to out-of-field dose “plateaus.” The orientation of the collimator has been shown to have a significant effect on out-of-field dose. Taylor *et al*.[51] found that preferentially aligning the craniocaudal axis of the patient with the x-plane (defined by the direction of jaw motion and effected by rotating the collimator appropriately) can reduce the out-of-field dose by up to an order of magnitude for a Varian 600C linac. In a study of out-of-field dose in pediatric radiotherapy, it was shown that alignment with the x-plane on other Varian linacs can also achieve a significant reduction in out-of-field dose. The percentage contribution to out-of-field dose (relative to central axis dose) due to collimator scatter generally differs amongst machines, as indicated by Figure 11.

#### The influence of treatment type

**Figure 12:**
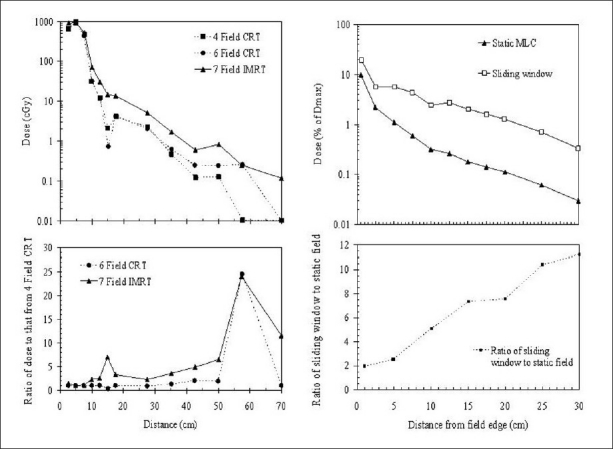
A comparison of IMRT with conformal (CRT) techniques [adapted from Wang and Xu (2008)].[45] The subplot is a ratio plot of the 6-field CRT and IMRT deliveries compared to the 4-field CRT delivery (monitor units were 1260, 1308 and 2850, respectively). Difference between achieving a 14×14-cm^2^ field with a static MLC or with a sliding-window technique [adapted from Sharma *et al*. (2006a)].[39] The subplot shows the ratio of the sliding-window case to the static case; achieving an equivalent field with the sliding window generates up to an order of magnitude more out-of-field dose

The nature of the treatment affects the out-of-field dose. Intensity-modulated radiotherapy (IMRT) is of particular interest in this regard,[57,62] as discussed earlier, because it typically involves a greater number of monitor units than other delivery methods. Wang and Xu[63] found that out-of-field doses are indeed significantly higher for an IMRT treatment than for conformal radiotherapy (CRT), as shown in Figure 12(a). Sharma *et al*.[39] showed that achieving an equivalent field size with a sliding field rather than a static MLC can result in an increase in out-of-field dose of up to an order of magnitude; see Figure 12(b). Hall and Wu[64] found that IMRT of prostate cancer rather than conventional radiotherapy resulted in double the risk of fatal secondary cancer (3% Sv^-1^ compared to 1.5% Sv^-1^). Kry *et al*.[55] found that 18-MV IMRT with a Varian unit resulted in a risk of fatal secondary cancer of 5.1% Sv^-1^, while the risk for 18-MV conventional radiotherapy was 1.7%.

#### Clinical implications and simple means of reducing out-of-field dose

There is strong evidence for radiation-induced cancer, even at low dose levels, such as those due to out-of-field dose from radiotherapy procedures.[4,5] The International Commission on Radiological Protection[6] does not assign specific dose limits to patients undergoing radiotherapy, precisely because it is the cell-killing function of ionizing radiation that is the desired effect, and restricting any dose may reduce the efficacy of the treatment. Few would argue with the notion that the curative effects of radiation therapy outweigh the potential detrimental consequences; nonetheless, there is clearly a need to maintain an awareness of out-of-field doses and the risks they pose to patients. This is of particular importance in certain contexts, such as the treatment of pediatric patients.

What has been demonstrated in the present work is that there are a range of contributors to out-of-field dose. As such, it may be possible — by careful selection of treatment parameters — to minimize out-of-field dose and the associated risks thereof. The day-to-day transferral of patients between linacs in a clinic is routine, because there is an understanding that a treatment on a different linac is equivalent provided the treatment plan is the same. For the in-field doses, this should indeed be the case, since linacs are individually commissioned and linac-specific data is incorporated into the treatment planning system (TPS). For this reason, anecdotal evidence suggests that oncologists and other clinicians are often surprised that there may be significant differences in out-of-field dose, and that in this regard treatments on different machines may not be identical. Treatment planning systems are generally commissioned using data that extend only centimeters beyond the field edge, with penumbra defined as 80% to 20% of the maximum dose for the field. Dose beyond this field is not intended to be incorporated into the overall dose calculation or to contribute to the inverse optimization procedure, and indeed it has been shown that treatment planning systems significantly miscalculate doses far from the primary field.[50]

In cases where out-of-field doses may be more critical, such as treatment of a pediatric patient or pregnant female, it is worthwhile considering the various influences on out-of-field dose. Where possible, attempts should be made to choose delivery parameters that result in an equivalent treatment to the targeted lesion but are likely to generate less out-of-field dose. A summary of parameters that influence out-of-field dose is given in Table 2.

**Table 1:** Excess relative risk (relative risk minus 1; ERR per Sv) of mortality for non-cancer diseases, identified for individual causes, shown with 90% confidence interval (CI). Based on life span study data[23]

*Non-cancerous disease*	*ERR (Sv-1)*	*90% CI*
Heart disease	0.17	0.08, 0.26
Stroke	0.12	0.02, 0.22
Respiratory disease	0.18	0.06, 0.32
Digestive disease	0.15	0.00, 0.32
Infectious disease	– 0.02	–0.2, 0.25
Other (non-blood) diseases	0.08	–0.04, 0.23
All non-cancer diseases	0.14	0.08, 0.2

Note that these are not strict rules, but merely general observations based on the data presented in the present review. It is strongly recommended that each institute / clinic undertake its own measurements of out-of-field dose using equipment available, and use these measurements to inform clinical decisions.

## Outlook

**Figure 13:**
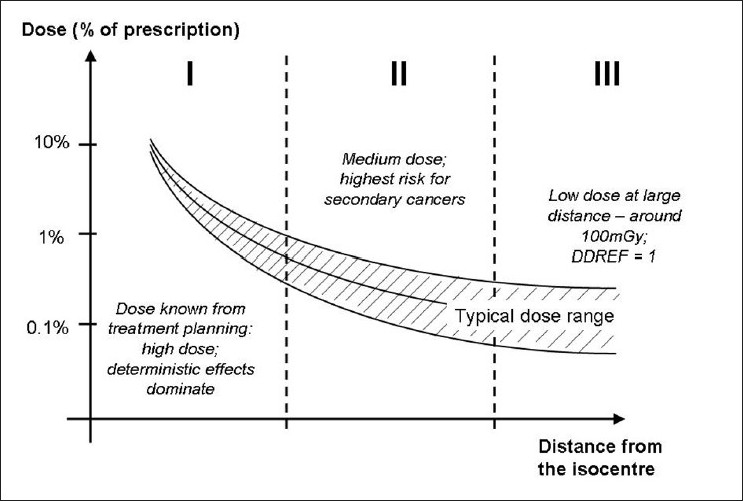
A conceptual illustration indicating 3 regimes of out-of-field dose. The high-dose region (where effects are likely to be deterministic) is typically calculated well by modern treatment planning systems. The dose region several centimeters beyond the penumbra (where stochastic effects are dominant) is best calculated by Monte Carlo methods. Far from the primary field (where low-risk stochastic effects are relevant), measurement of the leakage dose would best inform risk assessment — since it is relatively difficult to construct a Monte Carlo model that very accurately determines leakage dose at large distances

There is an increasing awareness of out-of-field doses and their corresponding long-term risks in radiotherapy. One can consider 3 broad regimes of out-of-field dose, illustrated in Figure 13. Close to the primary field, where deterministic effects are relevant, the treatment planning systems typically calculate doses well. Centimeters beyond the penumbra (where stochastic effects are the relevant risk), planning systems tend not to predict dose accurately, and a Monte Carlo model that accurately calculates collimator scatter and patient-scatter would be the preferable option for dose determination. Far from the primary field, where low-risk stochastic effects are relevant, measurement of the leakage dose would best inform risk assessment — since it is difficult and computationally-intensive to achieve accurate Monte Carlo predictions of leakage dose at large distances.

It is conceivable that the increasing efficacy of radiotherapy, which serves to increase patients’ lifetimes, will result in greater consideration of potential late effects, such as radiation-induced cancer. There are notable relationships between out-of-field dose and treatment parameters, which indicates that plan optimization in terms of out-of-field dose reduction is achievable.

**Table 2:** A concise overview of the various influences on out-of-field (OF) dose

*Parameter*	*Suggestions for reducing out-of-field dose*
Accelerator model	Philips and Varian linacs generate less OF photon dose than Siemens, though the Varian generates more neutron dose. Vertical waveguide inacs should be used in preference to horizontal waveguide (multiple-energy mode) linacs.
Collimator	Particularly on Varian machines, alignment of the craniocaudal axis with the x-plane of the collimators may result in reduction in OF dose of up to an order of magnitude. Collimator scatter varies between machines but becomes less important at large distances.
Energy	OF photon doses are generally lower in high-energy modes. High-energy modes generate neutron doses, but there is high uncertainty in neutron dose equivalents, and the actual increased risk due to neutrons may not be significant.
Treatment technique	Intensity-modulated radiotherapy generally results in significantly higher OF dose than conformal radiotherapy. Stereotactic radiotherapy generates similar OF doses far from the primary field, where leakage begins to dominate over scatter.
Shielding	The dominance of leakage over patient-scatter far from the primary field indicates shielding around the patient may reduce OF dose. Care must be taken that backscatter from shielding does not introduce additional OF dose.
